# Association between Noninvasive Fibrosis Markers and Cardio-Vascular Organ Damage among Adults with Hepatic Steatosis

**DOI:** 10.1371/journal.pone.0104941

**Published:** 2014-08-11

**Authors:** Giorgio Sesti, Angela Sciacqua, Teresa Vanessa Fiorentino, Maria Perticone, Elena Succurro, Francesco Perticone

**Affiliations:** Department of Medical and Surgical Sciences, University “Magna Graecia” of Catanzaro, Catanzaro, Italy; Bambino Gesu’ Children Hospital, Italy

## Abstract

Evidence suggests that advanced fibrosis, as determined by the noninvasive NAFLD fibrosis score (NFS), is a predictor of cardiovascular mortality in individuals with ultrasonography-diagnosed NAFLD. Whether the severity of histology (i.e., fibrosis stage) is associated with more pronounced cardiovascular organ damage is unsettled. In this study, we analyzed the clinical utility of NFS in assessing increased carotid intima-media thickness (cIMT), and left ventricular mass index (LVMI). In this cross-sectional study NFS, cIMT and LVMI were assessed in 400 individuals with ultrasonography-diagnosed steatosis. As compared with individuals at low probability of liver fibrosis, individuals both at high and at intermediate probability of fibrosis showed an unfavorable cardio-metabolic risk profile having significantly higher values of waist circumference, insulin resistance, high sensitivity C-reactive protein (hsCRP), fibrinogen, cIMT, and LVMI, and lower insulin-like growth factor-1 (IGF-1) levels. The differences in cIMT and LVMI remained significant after adjustment for smoking and metabolic syndrome. In a logistic regression model adjusted for age, gender, smoking, and diagnosis of metabolic syndrome, individuals at high probability of fibrosis had a 3.9-fold increased risk of vascular atherosclerosis, defined as cIMT>0.9 mm, (OR 3.95, 95% CI 1.12–13.87) as compared with individuals at low probability of fibrosis. Individuals at high probability of fibrosis had a 3.5-fold increased risk of left ventricular hypertrophy (LVH) (OR 3.55, 95% CI 1.22–10.34) as compared with individuals at low probability of fibrosis. In conclusion, advanced fibrosis, determined by noninvasive fibrosis markers, is associated with cardiovascular organ damage independent of other known factors.

## Introduction

Nonalcoholic fatty liver disease (NAFLD) is the most common cause of chronic liver disease in Western countries [1]–[2], encompassing a spectrum of conditions ranging from simple steatosis, to inflammatory steatohepatitis (NASH) with increasing levels of fibrosis and ultimately cirrhosis [3], [4]. While simple liver steatosis is regarded as a nonprogressive condition, NASH is a potentially harmful disorder associated with increased risk of liver-related morbidity and mortality [5]–[8]. Both NAFLD and NASH are strongly associated with a clustering of cardio-metabolic risk factors including obesity, hypertension, atherogenic dyslipidemia, lower plasma insulin-like growth factor-1 (IGF-1) levels, higher plasma inflammatory and hemostatic factors, insulin resistance, metabolic syndrome, endothelial dysfunction, impaired glucose tolerance, and type 2 diabetes [9]–[15]. Accordingly, NAFLD and NASH are both linked to an increased risk of incident cardiovascular events [6], [7], [16], [17].

Percutaneous liver biopsy is considered as the gold standard method for the assessment of hepatic fibrosis and inflammation severity in chronic liver disease but has several limitations, including invasiveness, complications, sampling variability, and costs [18]. In an attempt to overcome these problems, several noninvasive scoring indexes have been developed by combining clinical and serological variables that are capable to discriminate the presence or the absence of advanced fibrosis in subjects with NAFLD [19]–[23]. Recently, it has been reported that advanced fibrosis, as determined by the noninvasive NAFLD fibrosis score [20], is a significant predictor of mortality, mainly from cardiovascular causes, in individuals with ultrasonography-diagnosed NAFLD [24], [25].

The CATAnzaro MEtabolic RIsk factors (CATAMERI) study represents a well-designed cross-sectional study with a large sample size of Italian White adults [26]. In addition to the large number of anthropometric and cardio-metabolic variables, the CATAMERI study includes ultrasound data for NAFLD, carotid artery intima-media thickness (cIMT), and left ventricular mass (LVM) [13]–[15], [27], [28]. In the present study, we aimed to analyze the clinical utility of NAFLD fibrosis score in assessing cardiovascular organ damage including increased cIMT, and left ventricular hypertrophy (LVH) in a cohort of individuals with ultrasonography-diagnosed hepatic steatosis.

## Materials and Methods

The study group comprised 400 White individuals participating to the CATAMERI study, a cross-sectional study assessing cardio-metabolic risk factors in individuals carrying at least one risk factor including dysglycemia, overweight/obesity, hypertension, dyslipidemia, and family history for diabetes [26]–[28]. The protocol was approved by the local ethical committees (Comitato Etico Azienda Ospedaliera “Mater Domini”, Catanzaro, Italy), and written informed consent was obtained from all participants in accordance with principles of Helsinki Declaration.

Information regarding medical history, drug use, alcohol, and cigarette consumption were collected. Exclusion criteria included: history of malignant disease, gout, chronic gastrointestinal diseases associated with malabsorption, chronic pancreatitis, regular use of steatosis-inducing drugs, self-reporting alcohol consumption of >20 g/day, positivity for antibodies to hepatitis C virus (HCV) or hepatitis B surface antigen (HBsAg), absence of autoantibodies indicative of autoimmune hepatitis, Wilson’s disease, hemochromatosis, celiac disease, cholestatic liver disease, liver cirrhosis, and history of use of toxins or drugs known to induce liver damage. Clinical cardiovascular disease, including myocardial infarction, angina, heart failure, peripheral vascular disease, and stroke, was excluded on the basis of medical history, resting electrocardiogram, and echocardiographic assessments. All anthropometric and serological measurements were made in the morning after a 12-h fasting using standardized methods. Weight was measured in subjects in undergarments, height was measured by stadiometer, and body mass index (BMI) was calculated as body weight (kilograms) divided by the square of height (meters). Waist circumference was measured as the narrowest circumference between the lower rib margin and the anterior superior iliac spine. Brachial blood pressure was measured in the left arm of the supine subjects, after 5 min of quiet rest, with a digital electronic tensiometer (regular or large adult cuffs were used according to arm circumference). A minimum of three blood pressure readings were taken on three separate occasions at least 2 weeks apart, and the medians of these three values were used. A 75 g oral glucose tolerance test (OGTT) was performed with sampling for plasma glucose.

Intima–media thickness of the common carotid artery (cIMT) was measured by ATL HDI 3000 ultrasound system (Advanced Technology Laboratories, Bothell, WA) equipped with a 5 MHz linear array transducer as previously described [27]. Manual measurements were conducted in plaque-free portions of the 10-mm linear segment proximal to the carotid bulb. For each patient two measurements were performed bilaterally, and the values were averaged, to obtain the mean of IMT of the common carotid artery. Ultrasound study was performed by an experienced examiner who was unaware of the subjects’ clinical and laboratory findings. A value of IMT>0.9 mm was used as index of vascular atherosclerosis according to the 2013 Guidelines for the management of arterial hypertension released by the Task Force for the Management of Arterial Hypertension of the European Society of Hypertension (ESH) and of the European Society of Cardiology (ESC) [29].

Liver ultrasonography was performed in all participants by the same trained operator, who was blind to their clinical characteristics, using a Toshiba Aplio 50 ultrasound apparatus equipped with a 3.5-MHz linear transducer [13], [15]. Longitudinal, sub costal, ascending, and oblique scans were performed. The ultrasonographic criteria used to diagnose fatty liver included liver and kidney echo discrepancy, the presence of an increased liver echogenicity or “bright liver”, poor echo penetration into the deep portion of the liver, and vascular blurring either singly or in combination. A semi-quantitative ultrasound evaluation of the degree of steatosis was not available.

Echocardiographic assessments were performed by a single experienced examiner, who was blinded to the clinical and laboratory results of the study group. Tracings were taken with patients in a partial left decubitus position using a VIVID-7 Pro ultrasound machine (GE Technologies, Milwaukee, WI) with an annular phased array 2.5-MHz transducer. Only frames with optimal visualization of cardiac structures were considered for reading. The mean values from at least five measurements of each parameter for each patient were computed. Having the same experienced sonographer perform all studies in a dimly lit and quiet room optimized the reproducibility of measurements. In our laboratory, the intra-observer coefficients of variation (CVs) were 3.85% for posterior wall (PW) thickness, 3.70% for inter-ventricular septal (IVS) thickness, 1.50% for left ventricular internal diameter (LVID), and 5.10% for left ventricular mass (LVM). Tracings were recorded under two-dimensional guidance, and M-mode measurements were taken at the tip of the mitral valve or just below. Measurements of IVS thickness, PW thickness, and LVID were made at end-diastole and end-systole. LVM was calculated using the Devereux equation [30] and normalized by body surface area (LVM index [LVMI]). Partition values for LVH were taken with the cutoff value of 115 g/m^2^ for men and 95 g/m^2^ for women according to the 2013 Guidelines for the management of arterial hypertension released by the Task Force for the Management of Arterial Hypertension of the European Society of Hypertension (ESH) and of the European Society of Cardiology (ESC) [29].

### Analytical determinations

Glucose, triglycerides, total, low-density lipoprotein (LDL) and high-density lipoprotein (HDL) cholesterol concentrations were determined by enzymatic methods (Roche, Basel, Switzerland). Alanine aminotransferase (ALT) and aspartate aminotransferase (AST) levels were measured using the α-ketoglutarate reaction; gamma-glutamyltransferase (GGT) levels with the L-gamma-glutamyl-3-carboxy-4-nitroanilide rate method. Albumin concentration was determined with a Alb2 kit on a Cobas C6000 analyzer (Roche Diagnostics, Milan, Italy). High sensitivity C reactive protein (hsCRP) levels were measured by automated instrument (CardioPhase hsCRP, Milan, Italy). The erythrocyte sedimentation rate (ESR) was measured automatically by the stopped-flow technique in a capillary microphotometer (Alifax Test 1 System Polverara, Italy). An automated nephelometric technology using the BN II System analyzer (Siemens Healthcare, Italy) was employed to measure plasma fibrinogen concentration. Plasma insulin concentration was measured with a chemiluminescence-based assay (Immulite, Siemens, Italy), and total serum IGF-1 levels were determined by one-step sandwich chemiluminescence immunoassay (CLIA) after prior separation of IGF-I from binding proteins on the Liaison autoanalyzer (DiaSorin, Saluggia, Italy).

### Definitions

Glucose tolerance status was diagnosed according to the American Diabetes Association (ADA) criteria [31]: normal glucose tolerance (NGT) when fasting plasma glucose (FPG) was <5.6 mmol/l and 2 h post-load <7.8 mmol/l, isolated impaired fasting glucose (IFG) when FPG was 5.6–6.9 mmol/l and 2 h post-load <7.8 mmol/l, impaired glucose tolerance (IGT) when FPG was ≤6.9 mmol/l and 2-h post-load was 7.8–11.0 mmol/l, type 2 diabetes when FPG was ≥7.0 mmol/l and/or 2 h post-load was ≥11.1 mmol/l.

The NAFLD fibrosis score was calculated according to the following formula: −1.675+0.0373×age (years) +0.0943×BMI (kg/m^2^) +1.13×impaired fasting glycemia or diabetes (yes = 1, no = 0) +0.99×AST/ALT ratio −0.013×platelet (×10^9^/l–0.66×albumin (g/dl) [20]. Two cutoff points (>0.676 and <−1.455) were used to divide the subjects in three groups: low probability of fibrosis (<−1.455), intermediate probability of fibrosis (−1.455–0.676), and high probability of fibrosis (>0.676) [20]. The AST to platelet counts ratio (APRI) index was calculated as AST level (IU/L) divided by upper limit of AST (37 IU/L) and platelet counts (×10^9^/L), and finally multiplied by 10^2^ [19]. The BARD score was calculated by designating 0–2 points to the following parameters: BMI≥28 kg/m^2^ = 1 point, BMI<28 kg/m^2^ = 0 point; AST/ALT ratio≥0.8 = 2 points, AST/ALT ratio<0,8 = 0 points; diagnosis of type 2 diabetes mellitus = 1 point [22]. The FIB-4 index was calculated as age ([years]×AST [IU/L])/((platelets [10^9^/L])×(ALT [IU/L])^1/2^) [21]. The Fatty liver index (FLI) was calculated as (e 0.953×loge (triglycerides) +0.139×BMI +0.718×loge (GGT) +0.053×waist circumference – 15.745)/(1+ e 0.953×loge (triglycerides) +0.139×BMI +0.718×loge (GGT) +0.053×waist circumference – 15.745)×100 [32].

The metabolic syndrome was defined according to the criteria of the consensus statement released in 2009 [33]. Using this definition, a subject has metabolic syndrome if he or she meets three or more of the following criteria: 1) waist circumference >102 cm in men and >88 cm in women, 2) triglycerides ≥1.7 mmol/l or on drug treatment for elevated triglycerides, 3) HDL<1.03 mmol/L in men and <1.29 mmol/l in women or on drug treatment for reduced HDL, 4) blood pressure >130/85 mmHg or on antihypertensive drug treatment in a patient with a history of hypertension, and 5) fasting glucose ≥5.6 mmol/l. Individual 10-year CHD risk was estimated using the Framingham Heart Study prediction score sheet [34].

### Statistical analysis

Variables with skewed distribution including triglycerides, hsCRP, ESR, and fasting insulin were natural log transformed for statistical analyses. Continuous data are expressed as means ± SD. Categorical variables were compared by χ2 test. Anthropometric and metabolic differences between groups were tested after adjusting for gender and age using a general linear model with *post hoc* Bonferroni correction for multiple comparisons. A logistic regression analysis adjusted for several confounders was used to determine the association between the study groups and organ damage including LVH, and vascular atherosclerosis (IMT>0.9 mm). A two-tailed *P* value<0.05 was considered statistically significant. The ability of each noninvasive scoring index to detect individuals with organ damage was assessed by the area under the receiver operating characteristic (ROC) curve. The area under the ROC curve (AUC) was used as a measure of how well noninvasive scoring indexes identify LVH and vascular atherosclerosis. An area under ROC curve of 1.0 indicates perfect classification of subjects with high risk for organ damage, whereas 0.5 means that the classification is not better than chance. To determine whether the areas under ROC curve were significantly different, we used the method of Delong et al. [35]. All analyses were performed using SPSS software program Version 16.0 for Windows. A power analysis was conducted to determine the number of participants needed in this study using the G*Power software (http://www.gpower.hhu.de/). To achieve power of 95% (for α = 0.05) and an effect size *f* = 0.20, a sample size of 400 is required to detect a significant model (critical F-value = 3.02).

## Results

The clinical and biochemical features of the study group are described in Table 1. The mean age for the entire cohort was 53.7±10.7 years, with 43.2% being women. Of the 400 subjects examined, 146 (36.5%) had NGT, 47 (11.8%) had IFG, 86 (21.5%) had IGT, and 121 (30.2%) had type 2 diabetes. Metabolic syndrome was diagnosed in 287 (71.8%) individuals, and 261 (65.2%) subjects had hypertension treated with anti-hypertensive medications. A low probability of advanced liver fibrosis (NAFLD fibrosis score <−1.455) was found in 41% of the subjects, an intermediate probability of advanced liver fibrosis (NAFLD fibrosis score −1.455–0.676) was found in 50.5% of the subjects, and a high probability of advanced liver fibrosis (NAFLD fibrosis score >0.676) was found in 8.5% of the subjects. As expected by stratifying subjects according to the NAFLD fibrosis score, individuals classified as at high or intermediate probability of liver fibrosis were older (*P*<0.0001), had higher BMI (*P*<0.0001) and AST/ALT ratio (*P*<0.0001), lower platelet count (*P*<0.0001) and albumin levels (*P*<0.0001), and were more likely to have elevated fasting glucose (*P*<0.0001) and insulin (*P* = 0.009) or IFG/IGT/type 2 diabetes (*P*<0.0001) as compared with those at low probability of liver fibrosis. No differences in smoking habit were observed among the three groups of subjects. Subjects classified as at high or intermediate probability of liver fibrosis were more likely to have metabolic syndrome (*P*<0.0001) as compared with those at low probability of liver fibrosis. A higher proportion of individuals classified as at high or intermediate probability of liver fibrosis were treated with statins (*P*<0.0001) (Table 1). As consequence, individuals at high probability of fibrosis exhibited significantly lower levels of total and LDL cholesterol as compared with individuals at low probability of liver fibrosis (*P*<0.05 after adjustment for age and gender) (Table 1). In addition, a higher proportion of individuals classified as at high probability of fibrosis were treated with angiotensin-converting-enzyme (ACE) inhibitors, angiotensin receptor blockers and diuretics (Table 1).

**Table 1:** Anthropometric and biochemical characteristics of the study subjects stratified according to fibrosis risk score.

Variables	Whole studysubjects	Low probability offibrosis (<−1.455)	Intermediate probability offibrosis (−1.455–0.676)	High probability offibrosis (>0.676)	*P*
Number (Male/Female)	400 (227/173)	164 (89/75)	202 (115/87)	34 (23/11)	0.35
Age *(yrs)*	53.7±10.7	48.3±9.9	56.5±9.1^d^	63.6±9.7^d^	<0.0001
BMI *(kg/m^2^)*	32.3±6.1	30.5±5.6	33.2±5.9^b^	35.8±7.6^d^	<0.0001
Waist circumference (c*m*)	107±13	103±12	109±13^c^	114±16^d^	<0.0001
Current smokers No (%)	69 (17.2%)	33 (20.1%)	33 (16.3%)	3 (8.8%)	0.25
SBP *(mmHg)*	136±18	133±15	136±18	144±26	0.25
DBP *(mmHg)*	82±10	83±9	82±10	81±14	0.58
Fasting glucose (*mmol/l*)	6.44±2.78	5.55±2.16	6.77±2.72^c^	8.44±4.00^d^	<0.0001
2-h post-load glucose (*mmol/l*)	7.71±2.50	6.94±2.22	8.38±2.61^d^	9.16±2.44^d^	<0.0001
Fasting insulin *(pmol/l)*	104.18±62.51	97.23±62.51	111.12±62.51^b^	118.07±131.96^b^	0.009
Total cholesterol (*mmol/l*)	5.13±1.04	5.28±1.01	5.10±1.06	4.71±1.06^a^	0.08
LDL cholesterol (*mmol/l*)	3.11±0.91	3.26±0.85	3.03±0.91	2.72±1.04^a^	0.07
HDL cholesterol (*mmol/l*)	1.22±0.34	1.24±0.34	1.22±0.34	1.14±0.31^a^	0.08
Triglycerides (*mmol/l*)	1.67±0.82	1.58±0.78	1.72±0.82	1.79±82	0.42
ALT (*µkat/l*)	0.50±0.28	0.53±0.30	0.48±0.27	0.38±0.25^a^	0.04
AST (*µkat/l*)	0.42±0.23	0.38±0.15	0.40±0.17	0.55±0.37^b^	0.01
AST/ALT ratio	0.93±0.47	0.82±0.27	0.93±0.32^b^	1.48±1.10^d^	<0.0001
GGT (*µkat/l*)	0.60±0.47	0.60±0.45	0.57±0.42	0.60±0.50	0.59
Platelet count (*x10^9^/l*)	250±70	290±77	231±45^d^	175±46^d^	<0.0001
Albumin (*g/l*)	44.2±3.1	45.0±3.0	44.1±2.9^a^	41.6±2.9^d^	<0.0001
Fibrinogen (*µmol/l*)	9.17±2.12	8.61±1.76	9.50±2.29^b^	9.97±1.91^a^	0.03
hsCRP (*nmol/l*)	42.86±40.95	37.14±32.38	46.67±44.76^b^	58.10±55.24^c^	0.004
ESR (*mm/h*)	13±10	11±9	14±11^b^	20±13^c^	0.003
IGF-1 (*nmol/l*)	18.73±6.81	20.31±6.81	18.08±6.81^a^	15.20±5.24^c^	0.02
NFG/IFG/IGT/T2DM (No)	146/47/86/121	109/12/20/23	36/31/56/79^d^	1/4/10/19^d^	<0.0001
Metabolic syndrome No (%)	287 (71.8%)	94 (57.3%)	164 (81.2%)^d^	29 (85.3%)^d^	<0.0001
Therapy with statins No (%)	108 (27.0%)	30 (18.3%)	61 (30.2%)^b^	17 (50.0%)^c^	<0.0001
ACE inhibitor therapy No (*%*)	110 (27.5%)	40 (24.4%)	56 (27.7%)	14 (41.2%)^a^	0.13
Angiotensin receptor blocker therapy No (*%*)	97 (24.2%)	30 (18.3%)	56 (27.7%)^a^	12 (35.3%)^a^	0.03
Calcium channel blockers No (*%*)	84 (21.0%)	36 (22.0%)	39 (19.3%)	9 (26.5%)	0.59
Diuretics No (*%*)	96 (24.0%)	22 (13.4%)	57 (28.2%)^c^	17 (50.0%)^d^	<0.0001
Framingham risk score	6.8±4.7	5.0±4.6	7.9±4.2^d^	9.2±4.4^d^	<0.0001
Relative risk for 10-year CHD (%)	13±10	11±9	14±11^d^	20±13^d^	<0.0001

Data are means ± SD. Insulin, triglycerides, hsCRP, and ESR levels were log transformed for statistical analysis, but values in the table represent a back transformation to the original scale. Categorical variables were compared by χ^2^ test. *P* values refer to results after analyses with adjustment for gender and age. M = male; F = female; SBP = systolic blood pressure; DBP = diastolic blood pressure; LDL = low density lipoprotein; HDL = high density lipoprotein; hsCRP = high sensitivity C-reactive protein; ESR = erythrocyte sedimentation rate; IGF-1 = insulin-like growth factor-1; ALT = alanine aminotransferase; AST = aspartate aminotransferase; GGT = gamma-glutamyltransferase; ACE = angiotensin-converting-enzyme; NFG = normal fasting glucose; IFG = impaired fasting glucose; IGT = impaired glucose tolerance; T2DM = type 2 diabetes, CHD = coronary heart disease.

a *P*<0.05 vs. Low risk of fibrosis group.

b *P*<0.01 vs. Low risk of fibrosis group.

c *P*<0.001 vs. Low risk of fibrosis group.

d *P*<0.0001 vs. Low risk of fibrosis group.

As compared with individuals at low probability of liver fibrosis, individuals at high probability of fibrosis exhibited a worse cardio-metabolic risk profile having significantly higher values of waist circumference, hsCRP, fibrinogen, ESR as well as lower levels of HDL, and IGF-1 (Table 1). Individuals at high probability of fibrosis exhibited also a significantly higher Framingham risk score and had a higher relative risk of developing coronary heart disease over the next 10 years as compared with individuals at low probability of liver fibrosis (Table 1).

As compared with individuals at low probability of liver fibrosis, the individuals at intermediate probability of fibrosis exhibited an unfavorable cardio-metabolic risk profile having significantly higher values of waist circumference, hsCRP, fibrinogen, ESR, as well as lower levels of IGF-1 (Table 1). Individuals at intermediate probability of fibrosis exhibited also a significantly higher Framingham risk score and a higher relative risk of developing coronary heart disease over the next 10 years as compared with individuals at low probability of liver fibrosis (Table 1).

As compared with individuals at low probability of liver fibrosis, both individuals at high probability of fibrosis and individuals at intermediate probability of fibrosis exhibited higher value of cIMT (*P* = 0.026 and *P* = 0.031, respectively, after adjustment for age and gender using a general linear model with *post hoc* Bonferroni correction for multiple comparisons). By using a general linear model with *post hoc* Bonferroni correction for multiple comparisons to account for possible confounders, the differences in cIMT between individuals at low probability of liver fibrosis and those at high or intermediate probability of fibrosis remained statistically significant after additional adjustment for smoking history (*P* = 0.022 and *P* = 0.030, respectively) diagnosis of metabolic syndrome (*P* = 0.044 and *P* = 0.050, respectively) or for its individual components including waist circumference, blood pressure, HDL, triglycerides, and fasting glucose values (*P* = 0.034 and *P* = 0.046, respectively, Table 2), statin therapy (*P* = 0.044 and *P* = 0.050, respectively) or anti-hypertensive treatments (*P* = 0.041 and *P* = 0.046, respectively).

**Table 2:** Echocardiographic findings and carotid intima-media thickness measurements of the study subjects stratified according to fibrosis risk score.

Variables	Whole studysubjects	Low probability offibrosis (<−1.455)	Intermediate probability offibrosis (−1.455–0.676)	High probability offibrosis (>0.676)	*P*
cIMT (mm)	0.81±0.23	0.73±0.20	0.86±0.22^a^	0.99±0.23^a^	0.04
Left ventricular end-diastolicdiameter (cm)	5.0±0.5	4.9±0.4	5.1±0.5	5.3±0.7^a^	0.05
LVMI (g/m^2^)	120±34	111±32	124±32^a^	139±39^a^	0.04
LVH No (%)	243 (60.8%)	77 (47.0%)	137 (67.8%)	29 (85.3%)	<0.0001

Data are means ± SD. Categorical variables were compared by χ^2^ test. *P* values refer to results after analyses with adjustment for gender, age smoking history, waist circumference, blood pressure, HDL, triglycerides, and fasting glucose values.

cIMT = carotid artery intima-media thickness; LVMI = left ventricular mass index; LVH = left ventricular hypertrophy.

a *P*<0.05 vs. Low risk of fibrosis group.

A logistic regression model adjusted for gender, age, smoking history, and diagnosis of metabolic syndrome was used to compare the risk of individuals at high probability of fibrosis and individuals at intermediate probability of fibrosis to have vascular atherosclerosis, defined as cIMT>0.9 mm, as compared with individuals at low probability of fibrosis (the reference category). As shown in Table 3 (model 1), individuals at high probability of fibrosis had an 3.9-fold increased risk of having vascular atherosclerosis and individuals at intermediate probability of fibrosis had a 2.0-fold increased risk of having vascular atherosclerosis as compared with individuals at low probability of fibrosis.

**Table 3:** Logistic regression model comparing the risk of individuals at high, intermediate or low (the reference category) probability of liver fibrosis to have vascular atherosclerosis or left ventricular hypertrophy.

	OR	95% CI	*P*
**Model 1**			
Low probability of fibrosis (reference category)	1	–	–
Intermediate probability of fibrosis	2.01	0.99–4.06	0.051
High probability of fibrosis	3.95	1.12–13.87	0.03
**Model 2**			
Low probability of fibrosis (reference category)	1	–	–
Intermediate probability of fibrosis	1.74	1.08–2.80	0.02
High probability of fibrosis	3.55	1.22–10.34	0.02

Model 1, Odds ratios (95% CI) adjusted for age, gender, smoking history, and diagnosis of metabolic syndrome for vascular atherosclerosis.

Model 2, Odds ratios (95% CI) adjusted for age, gender, smoking history, and diagnosis of metabolic syndrome for left ventricular hypertrophy.

The area under the ROC (AUROC) curve was used to evaluate the accuracy of five noninvasive scoring indexes of liver damage i.e. NAFLD fibrosis score, FIB-4, BARD, APRI and FLI, and of the Framingham risk score in identifying individuals with vascular atherosclerosis. The AUC for NAFLD fibrosis score was significantly higher (0.732) as compared with the AUCs of APRI (0.541) (*P*<0.0001), BARD (0.594) (P = 0.0007), FIB-4 (0.666) (P = 0.05), and FLI (0.536) (P<0.0001) indexes, but did not differ as compared to the one of the Framingham risk score (Table 4).

**Table 4:** ROC curve analyses for detecting subjects with vascular atherosclerosis or left ventricular hypertrophy according to NAFLD fibrosis score, FIB-4, BARD, APRI and FLI noninvasive scoring indexes of liver damage, and Framingham score for 10-year CHD risk.

ROC curve analyses for detecting subjects with vascular atherosclerosis				
	AUC	SE	95% CI	*P* *
NAFLD fibrosis score	0.732	0.0375	0.674 to 0.785	–
APRI	0.541	0.0445	0.477 to 0.604	<0.0001
BARD	0.594	0.0433	0.531 to 0.656	0.0007
FIB4	0.666	0.0389	0.605 to 0.724	0.05
FLI	0.536	0.0458	0.472 to 0.599	<0.0001
Framingham score for 10-year CHD risk	0.730	0.0326	0.668 to 0.780	0.3
**ROC curve analyses for detecting subjects with left ventricular hypertrophy**				
	**AUC**	**SE**	**95% CI**	***P*** *
NAFLD fibrosis score	0.702	0.0261	0.654 to 0.747	–
APRI	0.504	0.0301	0.453 to 0.555	<0.0001
BARD	0.598	0.0277	0.548 to 0.647	0.001
FIB4	0.642	0.0284	0.592 to 0.690	0.006
FLI	0.511	0.0295	0.460 to 0.562	<0.0001
Framingham score for 10-year CHD risk	0.710	0.0269	0.663 to 0.754	0.33

*P<0.05 vs. NAFLD fibrosis score.

As compared with individuals at low probability of liver fibrosis, both individuals at high and at intermediate probability of fibrosis exhibited higher value of LVMI (*P* = 0.025 and *P* = 0.050, respectively, after adjustment for age and gender using a general linear model with *post hoc* Bonferroni correction for multiple comparisons). By using a general linear model with *post hoc* Bonferroni correction for multiple comparisons to account for possible confounders, the differences in LVMI between individuals at low probability of liver fibrosis and those at high or intermediate probability of fibrosis remained statistically significant after additional adjustment for smoking history (*P* = 0.024 and *P* = 0.049, respectively), diagnosis of metabolic syndrome (*P* = 0.027 and *P* = 0.050, respectively) or for its individual components including waist circumference, blood pressure, HDL, triglycerides, and fasting glucose values in addition to age and gender (*P* = 0.028 and *P* = 0.049, respectively, Table 2), statin therapy (*P* = 0.018 and *P* = 0.050, respectively) or anti-hypertensive treatments (*P* = 0.027 and *P* = 0.050, respectively).

A higher proportion of individuals classified as at intermediate or high probability had left ventricular hypertrophy (LVH), defined as LVMI>115 g/m^2^ for men and >95 g/m^2^ for women (29), as compared with individuals at low probability of fibrosis (*P*<0.0001) (Table 2). A logistic regression model adjusted for age, gender, smoking history, and diagnosis of metabolic syndrome was used to compare the risk of individuals at high or at intermediate probability of fibrosis to have LVH as compared with individuals at low probability of fibrosis (the reference category). As shown in Table 3 (model 2), individuals at high probability of fibrosis had a 3.5-fold increased risk of having LVH, and individuals at intermediate probability of fibrosis had a 1.7-fold increased risk of having LVH as compared with individuals at low probability of fibrosis.

The AUROC curve was used to evaluate the accuracy of the five noninvasive scoring indexes of liver damage and of the Framingham risk score in identifying individuals with LVH. The AUC for NAFLD fibrosis score was significantly higher (0.702) as compared with the AUCs of APRI (0.504) (*P*<0.0001), BARD (0.598) (P = 0.001), FIB-4 (0.642) (P = 0.006), and FLI (0.511) (P<0.0001) indexes, but did not differ as compared to the one of the Framingham risk score (Table 4).

## Discussion

It is increasingly recognized that both NAFLD and NASH are associated with a clustering of cardio-metabolic disorders including metabolic syndrome and abnormal glucose homeostasis, and predict the development of cardiovascular diseases [9]–[18]. There is also evidence supporting the notion that adverse clinical outcomes are more frequent in patients with NASH rather than in individuals with simple liver steatosis [6]–[8], [16]. A number of noninvasive scoring indexes combining clinical and biochemical variables have been developed aimed at identifying advanced fibrosis in subjects with NAFLD [19]–[23]. Using one of these liver fibrosis scores, it has been reported that advanced fibrosis is associated with increased risk of chronic kidney disease, and cardiovascular mortality in individuals with NAFLD [36], [24], [25]. These observations coupled with the accessibility of a carefully characterized cohort of adult individuals have provided the rationale for examining the relationship between advanced liver fibrosis, as determined by the NAFLD fibrosis score [20] in individuals with ultrasonography-diagnosed NAFLD and subclinical cardiovascular organ damage encompassing increased cIMT, and left ventricular hypertrophy (LVH). In the present cross-sectional study, we report that individuals with high probability of advanced liver fibrosis have increased cIMT, and LVMI, two reliable markers of subclinical organ damage, which predict development of cardiovascular events [37]–[39] as compared with individuals at low probability of liver fibrosis. These associations did not change after adjusting for several potential confounders including age, gender, smoking history, and diagnosis of metabolic syndrome. These data are consistent with those of a previous study showing that cIMT is strongly associated with the severity of liver histopathology in 85 subjects with biopsy-proven NAFLD [40]. Echocardiographic features of increased LV mass have been reported in adults with NAFLD [41], but its association with more advanced form of liver damage is a novel finding of the present study. Notably, the accuracy assessed by the areas under the ROC curve of the NAFLD fibrosis score in detecting subjects with vascular atherosclerosis or left ventricular hypertrophy, was the highest as compared with different noninvasive scoring indexes of liver fibrosis comprising APRI, BARD, and FIB-4, and was similar to that of the Framingham risk score.

The biological mechanism(s) by which NAFLD and NASH may contribute to subclinical cardiovascular damage are still unsettled, and their identification is beyond the scope of this study. The strong association between NAFLD/NASH, visceral obesity, insulin resistance, and metabolic syndrome make it extremely difficult to pinpoint the precise causal relationships underlying the increased risk of cardiovascular disease among individuals with NAFLD. However, putative underlying mechanisms linking NAFLD/NASH to the development and progression of cardiovascular disease may include visceral adipose tissue expansion, insulin resistance/hyperinsulinemia, endothelial dysfunction, chronic inflammation, hypercoagulability, and impaired IGF-1 production [9]–[16]. Interestingly, we found that individuals with high or intermediate probability of advanced liver fibrosis have an unfavorable cardio-vascular risk profile characterized by an increase in visceral adiposity, insulin resistance, inflammatory and pro-coagulant biomarkers such as hsCRP, erythrocyte sedimentation rate, and fibrinogen as well as lower levels of circulating IGF-1.

Overall, our findings may have important clinical implications. Since subjects with NASH may have a poor prognosis, it is important to identify individuals with higher probability of liver fibrosis so they can be subjected to further invasive and noninvasive investigations aimed both at preventing progression of liver disease, and development of cardiovascular complications.

The present study has several strengths, including the inclusion of both sexes, the relatively large sample size with detailed anthropometric, clinical, and cardio-metabolic variables, the strict quality control of ultrasound studies (liver, heart, and carotid artery) performed by experienced examiners who were blinded to the subjects’ clinical and laboratory findings, the centralized assays of biochemical variables, the use of restrictive *post hoc* Bonferroni test to correct for multiple comparisons, and the exclusion of confounding conditions characterized by elevation in liver enzymes such as heavy drinking, positivity for antibodies to HCV or HBsAg and cirrhosis. Nevertheless, some limitations should be acknowledged in the interpretation of our results. First, the diagnosis of hepatic steatosis was based on ultrasonography rather than on invasive methods such as liver biopsy or expensive and time-consuming non-invasive methods such as proton magnetic resonance spectroscopy or computed tomographic scanning. Although ultrasonography is the most common method of diagnosing for moderate to severe forms of hepatic steatosis in clinical practice, its sensitivity is suboptimal when hepatic fat infiltration of the liver is <30%. However, individuals of our cohort had normal or only mildly elevated serum liver enzymes and, therefore, liver biopsy may not be an appropriate investigation for many of them. A second limitation of our study is that all biochemical variables, including plasma glucose levels, were measured once. Although such an approach is common in most large epidemiological studies, these measures are subject to intra-individual variability, and this may have introduced some imprecisions in the classification of subjects into glucose tolerance groups. Furthermore, the information on alcohol intake was self-reported by participants, thus the true daily alcohol consumption may have been underestimated. Next, to avoid an unpredictable modification of the characteristics of the original sample, we chose not to exclude the subjects treated with lipid-lowering and anti-hypertensive therapies, which may attenuate inflammation-related markers, and subclinical cardio-vascular organ damage. However, adjusting for medication intake did not affect the results. Furthermore, our cohort comprises outpatients recruited at a referral university hospital, representing individuals at risk for cardio-metabolic disease, and, therefore, our results may not necessarily be extendible to the general population. Additionally, all participants to the present study were White, and whether these observations can also be extended to nonwhite ethnic groups remains to be determined. Finally, because of the cross-sectional design of the study, the present findings reflect only an association with prevalent and not incident subclinical cardio-vascular organ damage, and therefore no definitive cause and effect relationship can be inferred. Therefore, the present data should be considered hypothesis generating and requiring confirmation by further prospective studies in order to validate the benefit of noninvasive scoring indexes for predicting cardiovascular complications in subjects with hepatic steatosis.
